# Professorial Appointments

**Published:** 1850-08

**Authors:** 


*Professorial Appointments.*—Professorial changes continue to be the order of the day.

Prof. N. CHAPMAN, who has for a quarter of a century or more, occupied the chair of Practical Medicine in the University of Pennsylvania, has resigned, and Prof. GEORGE B. WOOD has been appointed to fill his place. Prof. WOOD has heretofore occupied the chair of Materia Medica. Joseph Carson, M. D., has been selected to fill the chair made vacant by the transfer of Prof. W.

Prof. DICKSON, late of the University of the city of New York, has been reinstalled in the Professorship formerfy held by him in the Medical College of Charleston, S. C.

Prof. BELLINGER has resigned the chair of Surgery in the latter institution, and Prof. GEDDINGS transferred from the chair of Practical Medicine, to fill his place. It is stated that Dr. DETMOLD, of the city of New York, has been appointed to fill the chair vacated by the resignation of Prof. DICKSON.

In the Medical College of Ohio (at Cincinnati,) all the chairs have recently been declared vacant by the Trustees. Drs. MUSSEY and LAWSON have been re-appointed, and Dr. RIEVE, (a new appointment) appointed to the chair of obstetrics. Dr. DRAKE, (as we are informed,) declines a re-appointment, and several of the chairs in that institution remain to be filled.

The foregoing was in type for our last number, and excluded by an overplus of editorial matter. Several changes have occurred since the article was written.

Dr. John Bell, of Philadelphia, has been appointed Professor of Practical Medicine in the Medical College of Ohio ; and Dr. T. O. Edwards, Professor of Materia Medica and Medical Jurisprudence in the same institution. Dr. Bell is the American author of Bell & Stokes' Practice of Medicine, by which, together with numerous other publications, he has earned a widely extended, and high reputation. He has also for many years been accustomed to give medical lectures, although not before, that we are aware of, connected with any collegiate institution. He will reside hereafter at Cincinnati.

Dr. Edwards is known to the profession from his connection with the passage of the law regulating the importation of drugs, being at that time a member of Congress.

Prof. Dudley, of the Transylvania Medical College, Lexington, Ky., has recently resigned the chair of surgery in that institution, which he has occupied with distinguished ability for many years. As an operating surgeon, especially in the operation of lithotomy, Prof. D. has a world-wide reputation. He has undoubtedly performed this operation a greater number of times, and with less mortality than any other surgeon living, or dead.

Prof. Mott, of the University of the city of New York, and the newly appointed Professor, Dr. Detmold, have recently resigned their places. The New York Medical Gazette informs its readers that Dr. Mott, being in Europe, immediately sent in his resignation on being apprized of the appointment of Dr. Detmold, to which he was opposed. Whereupon Dr. Detmold also resigned, and both resignations were accepted. We learn from the same source that applications are invited to fill the two chairs made vacant by these resignations.

